# Four Most Pathogenic Superfamilies of Insect Pests of Suborder Sternorrhyncha: Invisible Superplunderers of Plant Vitality

**DOI:** 10.3390/insects14050462

**Published:** 2023-05-13

**Authors:** Volodymyr V. Oberemok, Nikita V. Gal’chinsky, Refat Z. Useinov, Ilya A. Novikov, Yelizaveta V. Puzanova, Roman I. Filatov, Nanan J. Kouakou, Kra F. Kouame, Kouadio D. Kra, Kateryna V. Laikova

**Affiliations:** 1 Department of Molecular Genetics and Biotechnologies, Institute of Biochemical Technologies, Ecology and Pharmacy, V.I. Vernadsky Crimean Federal University, 295007 Simferopol, Crimea; 2 Nikita Botanical Gardens—National Scientific Centre, Russian Academy of Sciences, 298648 Yalta, Crimea; 3 Centre National de Floristique, Université Félix Houphouët-Boigny, Abidjan 01 BP V 34, Côte d’Ivoire; 4 Biology Laboratory and Animal Cytology, Université Nangui Abrogoua, Abidjan 02 BP 801, Côte d’Ivoire

**Keywords:** suborder Sternorrhyncha, plant protection, chemical insecticides, olinscides, sustainable development, climate change, insecticide resistance

## Abstract

**Simple Summary:**

Changing environmental parameters with the development of global warming and the growing anthropogenic influence lead to the spread of insect pests in new habitats, abundant in their host plants. While remaining visually undetected, representatives of the hemipteran insect pests in the suborder Sternorrhyncha cause large-scale economic losses. In this review, we consider the main pathogenic superfamilies of the suborder and present new methods of dealing with them that meet the requirements for modern insecticides and take into account the need for the development of insecticides that do not cause global warming. We solve the problem of environmental pollution caused by modern insecticides by proposing the use of oligonucleotide insecticides based on conservative fragments of genomes of insect pests which slow down the emergence of resistance to the applied insecticides. Our proposed approach opens up new horizons for both safe and effective insect pest control.

**Abstract:**

Sternorrhyncha representatives are serious pests of agriculture and forestry all over the world, primarily causing damage to woody plants. Sternorrhyncha members are vectors for the transfer of a large number of viral diseases, and subsequently, the host plant weakens. Additionally, many are inherent in the release of honeydew, on which fungal diseases develop. Today, an innovative approach is needed to create new and effective ways to control the number of these insects based on environmentally friendly insecticides. Of particular relevance to such developments is the need to take into account the large number of organisms living together with insect pests in this group, including beneficial insects. Practically without changing their location on their host plant, they adopted to be more invisible and protected due to their small size, symbiosis with ants, the ability to camouflage with a leaf, and moderately deplete plants and others, rarely leading them to death but still causing substantial economic loss in the subtropics and tropics. Due to the lack of presence in the literature, this review fills in this pesky spot by examining (on the example of distinct species from four superfamilies) the characteristic adaptations for this suborder and the chemical methods of combating these insects that allow them to survive in various environmental conditions, suggesting new and highly promising ways of using olinscides for plant protection against Sternorrhyncha members.

## 1. Introduction

Insects have hampered agriculture: they consume crops, spread diseases, and damage infrastructure [1,2]. In total, insect pests reduce agricultural yields worldwide by 20–32% before harvest and following harvest [3]. Some representatives of Sterrnorhyncha cause yield losses of up to 100% [4,5,6,7]. For example, papaya mealybug *Paracoccus marginatus Williams and Granara de Willink* (Hemiptera: Pseudococcidae) infestation is a significant driver of papaya yield losses, with losses up to 75% in Tanzania [8] and 57% in Kenya [9]. Whiteflies cause complete tomato yield losses because they vector several tomato begomoviruses, including tomato golden mosaic virus, that subsequently cause great losses in yields or otherwise severely damage the crops in Brazil [4,5,6,7]. The sugarcane aphid, *Melanaphis sacchari* Zehntner (Hemiptera: Aphididae), has presently become to be an economically imperative pest of sorghum in Mexico and more than 17 states of the United States [10]. Losses of up to 50–70% of yield due to infestation by *M. sacchari* have been reported [11]. Some estimates suggest that white mango scale, *Aulacaspis tubercularis Newstead* (Hemiptera: Diaspididae), can cause imperative economic losses within the Spanish organic mango plantations, which may surpass 40% in late-ripening cultivars [12]. In fact, the United States and China, as two largest food-producing countries, exhibit the biggest losses from invasive insects, including some Sternorrhyncha species, *Aphis gossypii Glover*, *Aphis fabae Scopoli,* and *Aonidomytilus albus Cockerell* [13]. Several other insect pests from Sternorrhyncha threaten commercial forestry, degrade plant biodiversity, and increase tree mortality and associated increases in greenhouse gas emissions, thus hampering climate change mitigation [14,15,16].

This insect order has exploited diverse food sources and habitats during their course of evolution that lasts more than 300 million years. Representatives of Sternorrhyncha possess highly specialized morphological and physiological adaptations [17]. Hemiptera is among the biggest insect orders (along with Diptera, Coleoptera, Hymenoptera, and Lepidoptera). Hemipterans represent around 7% of metazoan diversity and are the most diversified and species orders among all insects, being the largest non-holometabolous order of insects [17,18]. More than 110,000 species are already described in the Hemiptera order and there are nearly 320 extant and extinct families in it [19,20,21,22]. For the moment there are six suborders in the order Hemiptera [19]: Sternorrhyncha (jumping plantlice, modern aphids, whiteflies, scale insects, and their extinct relatives), Fulgoromorpha (planthoppers), Cicadomorpha (cicadas, leafhoppers, froghoppers, treehoppers, and number of extinct groups), Heteroptera (true bugs), Coleorrhyncha (moss bugs), and extinct Paleorrhyncha (archescytinoids) [17], holding the highest number among all insect orders.

Representatives of the Sternorrhyncha, which comprise about 18,700 recently described species, are tiny phytophages of great economic and ecological importance and usually recognized as superfamilies: Coccoidea (coccoids scale or insects), Aleyrodoidea (white flies), Psylloidea (psylloids or jumping plant-lice, often collectively called psyllids), and Aphidoidea (aphids or aphidoids) [15,17,19,22]. The name “Sternorrhyncha” (from the Greek “sternon” meaning chest and “rhynchos” meaning nose or snout) explains the location of the mouthparts between the bases of the front legs on the underside of the insect, but the mouthparts are sometimes lacking in the adult [15].

For plant pathogens, some Sternorrhyncha species act as vectors. Aphids transmit more than 150 plant viruses in agricultural systems [22,23,24]. Aphids transmit viruses from a few families, counting Potyviridae, which is the biggest plant-infecting RNA virus family [25]. Potyviruses, along with their aphid vectors, cause significant agrarian misfortunes all through the world [26]. *Bemisia tabaci* Gennadius (Hemiptera: Aleyrodidae) vectors plant viruses causing the most severe crop damage. Over 350 plant virus species from five genera are transmitted by *B. tabaci*: ipomoviruses (family Potyviridae, begomoviruses (family Geminiviridae), torradoviruses (family Secoviridae), criniviruses (family Closteroviridae), and some carlaviruses (family Betaflexiviridae), causing complete yield losses for important industrial and food crops in particular times and places [4,5,6,7,27]. Most of the viruses transmitted are begomoviruses [6,28,29,30,31].

As a part of their biology, some members of Sternorrhyncha suborder release honeydew (a sticky sugary excreta) that may contaminate foliage. Honeydew is a substrate for the growth of black sooty mold fungi species of *Cladosporium*, *Fumago*, and *Capnodium* that can reduce plant vigor and impede photosynthesis. Ants are also attracted by honeydew and often protect the Sternorrhyncha members from their natural enemies, especially parasitic and predatory insects [15,22].

One of the key factors of Sternorrhyncha species that cause problems in agricultural systems is their propensity for invasion. They are invisible superplunderers of plant vitality closely associated with their host plants and easily going undetected through quarantines. Sternorrhyncha members camouflage and often go unrecognized because they are small and cryptic. Approximately ten new scale insect species have been introduced to the United States each decade, and five of these have become established as insect pests [32].

The “pestiness” of Sternorrhyncha is expensive. For example, in Florida alone, the Asian citrus psyllid, *Diaphorina citri Kuwayama*, as a vector for bacteria *Candidatus Liberibacter* species, causing citrus greening, causes losses that have been estimated to exceed US$ 3.6 billion in a 5 year period [33,34,35], and the citrus industry worldwide is in serious danger because of its spread [36]. The worldwide cost associated with invasive insects is evaluated at US$ 77 billion per year, proportionate to the total cost for all goods and services and health (US$ 7 billion per year), of which US$ 5–6 billion per year comes from controlling some Sternorrhyncha species in regions of South and North America (*Adelges piceae*—US$ 1.5 billion, *Maconellicoccus hirsutus* Green—US$ 1.3 billion, *B*. *tabaci*—US$ 1 billion, *Bemisia argentifolii* Bellows and Perring—US$ 820 million, *Adelges tsugae* Annand—US$ 330 million) [1,37]. Today, world trade enhances connectedness and globalization, leading to an increase of threat of invasive species because of their distribution in countries where they were absent before and where relevant measures for their control are not properly developed [13,38].

Both conserving biodiversity and maintaining economic productivity, constitute a global challenge of this century while meeting food requirements of the world [1]. Tragically, the utilization of the lion’s share of pesticides postures a threat to the characteristic environment [39,40,41], and their inappropriate utilization leads to pesticide buildups in crops and nourishment items [42]. For human health, primary and derived agricultural products are the most real source of pesticide residues that are found in all agroecosystems [43,44]. Poorly tracked pesticide residues replenish concentrations of organic xenobiotics, poisoning ecosystems [45]. Different human health-related concerns are related with pesticides, from short-term impacts such as migraines and queasiness to inveterate impacts such as different cancers, birth defects, infertility, and endocrine disturbance [46,47,48,49]. Children are more impacted by short-term and unremitting exposure to pesticides [50]. As a result of expanding scientific evidence on the impact of pesticides, actions have been taken around the world, such as within the EU to decrease the risks related with pesticide utilization. In this area, Directive 2009/128/EC110 regulates EU policy priorities. This record, among others, obliges part-states to create national activity plans to decrease the risks related with the utilization of pesticides and the effect of their utilization on human well-being and the environment, and to advance the improvement and application of coordinated bug administration and elective approaches or strategies to diminish reliance on the utilization of pesticides [51]. The greenhouse gas emissions related with pesticide applications against intrusive species constitute an environmental cost that has generally gone unrecognized [52]. Currently, to extend the sustainability of the EU nourishment supply chains, beneath the European Green Bargain, the use of pesticides (basically within the rural division) is to be decreased by 50 percent by 2030 [53]. Recent findings show that human-related activities, such as the use of pesticides and other chemicals, have contributed tremendously to the effects of climate change via the emission of greenhouse gases from the production of chemicals [54]. Carbon dioxide (CO_2_), nitrous oxide (N_2_O), and methane (CH_4_) are the primary greenhouse gases that contribute to global warming [52,55]. Therefore, it is important when attempting to control insect pests for whom chemical control is contraindicated, especially those whose natural predators are sparse, to find and use effective biopesticides that present a low risk that pests will develop resistance and few, if any, toxic effects on the environment and human health [56].

In this review, we examine the characteristic adaptations for the Sternorrhyncha species from the superfamilies Psylloidea, Aleyrodoidea, Aphidoidea, and Coccoidea, which are of ecological and economic importance around the world, the chemical methods used to control these insects, and propose the use of well-tailored and affordable oligonucleotide insecticides (olinscides) or DNA insecticides [57] with a zero (or minimal) carbon footprint for insect pest control. In addition, when studying the literature on this issue, we did not find extensive reviews on the biology of Sternorrhyncha or plant protection against them. In this review, we are pursuing, among other things, the goal of filling this gap in the scientific literature that concerns one of the most serious pests of agriculture and forestry in the world.

## 2. Superfamily Coccoidea

Nearly 8000 species of plant-eating hemipterans from 32 families make up the superfamily Coccoidea [58,59,60]. As adapted insect pests, they exhibit several highly specific morphological, genetic, and biological characteristics. Their remarkable sexual dimorphism features two-winged adult males without functional mouthparts and adult females that resemble nymphs and only live a few days. Body segmentation and the typical decrease of female appendages and the production of wax by specialized glands make the (often sessile) females cryptic and rarely noticeable in the host plant’s microhabitats [61,62,63]. Typically, the female has three to four instars while the male has five instars [32]. In most cases of dissemination, these insects are often crawlers (first-instar nymphs) [64]. Aside from their own powers of locomotion, they may be distributed by birds, insects, and occasional long way dispersion by wind. Additionally, when they come into contact with a plant, they spread to other plants, and ants that tend the crawlers may transfer them from one plant to another [65,66].

Some scale insects are serious plant pests, especially for agricultural perennials. Nut and fruit trees, forest or plantation trees, greenhouse plants, woody ornamentals, houseplants, and occasionally even sugarcane and lawn grass are among the things they can harm. Pests are typically oligophagous or polyphagous [58]. These insects can be found on many sections of their hosts, where they may infest leaves, twigs, branches, and roots; some of them even dwell inside plant domatia [61,67]. It should be noted that low population sizes of insect pests might make them notoriously challenging to find during quarantine inspections [58]. Therefore, the detection of new infestations is frequently postponed until populations have multiplied, or the symptoms of the plants are obvious. Depending on the type of scale, the initial behaviors that damage the plant are sap sucking and ingesting from almost any point on the plant, as well as physical penetration of stylets and injection of frequently toxic saliva into plant tissues [61,64]. Except for Diaspididae, which feed on parenchyma and create enormous amounts of honeydew rich in sugars that can be used by ants and foster mutualistic interactions, the majority of scale insect species are phloem feeders [63].

Although the Diaspididae, Coccidae, and Pseudococcidae families have the most species, there are a few significant pests that come from other families, including the Eriococcidae, Asterolecaniidae, and Monophlebidae (especially polyphagous *Icerya* species) [58,63,67,68,69].

Among the family Monophlebidae Morrison, 1928 (Hemiptera: Coccomorpha), it stands out, especially the genus *Icerya* Signoret, 1875, of the tribe Icerini Cockerell, 1899 [70]. There are 37 species widely distributed in the world [71] that belong to this genus, and they are referred to as “fluted scales” because the ovisac has a fluted appearance (egg bag) [72]. The female adult of these insects secretes an ovisac when she matures, which is their defining characteristic [37,73]. The huge, fluted ovisac, which is usually two to five times longer than the body, is the most striking characteristic. There are roughly 1000 red eggs in the ovisac. Depending on the temperature, eggs hatch into nymphs within a few days or up to two months. The major dispersal stage, which is the most harmful to the health of the plant, can be spread by wind, crawling to other plants, or even by other animals acting as vectors [73]. Worldwide, several *Icerya* species are polyphagous and eat landscape plants and commercially significant fruit trees [37,74,75]. Due to them, they pose a significant threat of invasion and a great potential for causing economic harm to the invaded regions [76]. Some *Icerya* species may spread and become harmful plant pests when introduced to new environments without the proper natural enemies [77]. For example, *Icerya aegyptiaca* Douglas in the Ryukyu Islands (Japan) [78] and *Icerya seychellarum* Westwood in Italy [79]. With their penetrating sucking mouthparts, *Icerya* species essentially suck plant juice, resulting in yellow leaves, deciduous leaves and fruits, withered shoots, and decreased tree vigor. They also exude honeydew, which coats the leaf surface, causes sooty blotches, and affects photosynthesis and decorative esteem [76].

*Icerya purchasi* Maskell is one of the most significant *Icerya* species with a significant invasive potential. *I. purchasi*, also known as the cottony cushion scale, is a widespread plant pest that is endemic to Australia and New Zealand and is reported to harm over 200 distinct plant species [73]. It is a pest of several ornamentals and crops, such as *Citrus reticulata* Blanco, *Artocarpus heterophyllus* Lam., *Magnolia denudate* Desr., *Ficus altissima* Blume, and *Pittosporum tobira* (Thunb.) W.T.Aiton [37]. The most damage occurs when the immature stages of the scale feed on the leaves, where they cluster in rows along the midribs and veins, and on the smaller twigs. This results in fruit loss, defoliation, and diminished tree health [80]. Additionally, this pest was unintentionally introduced into other nations; as a result, it is now present in 126 nations worldwide [71]. The uncontrolled infestation of *I. purchasi* has had a severe effect on the pomiculture and horticulture industries and the endemic fauna of the Galapagos Islands [81]. Moreover, the damage it causes to host plants leads to the extinction of Lepidoptera species that feed on them, such as *Semiothisa cerussata* Herbulot, *Platyptilia vilema* Landry, and *Tebenna galapagoensis* Heppner and Landry [82,83]. In Turkiye, *I. purchasi* has caused extensive damage to cherry laurel (*Prunus laurocerasus* L.) orchards located in the Black Sea region [84] and mimosa plants (*Acacia dealbata* Link) in Artvin [85]. They are pests of numerous ornamentals and crops in Oman, including *Punica granatum* L., *Juglans regia* L., *Ziziphus spina-christi* L., *Ficus carica* L., *Acacia sp.* Mill., *Nerium oleander* L., and others [86].

Organophosphates and petroleum oils are used to control this pest, and although buprofezin is effective on young nymphs, it fails to affect the adult pests [85]. At the same time, the demand for insecticide applications has skyrocketed as insect pests have become more resistant to existing pesticides [87]. While the use of a predator species, *Rodolia cardinalis* Mulsant (Coleoptera: Coccinellidae), has shown considerable potential in the control of cottony cushion scale populations [79,81,83,84,85], the activity of natural enemies is diminished by the blind use of broad-spectrum insecticides by farmers, since this kills both predators and prey; in addition, these insecticides have adverse effects on the environment [85,88,89].

Today, one of the most efficient and targeted techniques for eradicating insect pests from the Sternorrhyncha suborder is the use of olinscides [90,91,92,93,94]. Our findings indicate that ribosomal gene-based antisense oligonucleotide sequences are interesting candidates for use as olinscides. In the cells of living organisms, mRNA is contained in approximately 5% of all cellular RNA, while ribosomal RNA reaches up to 80% in proportion and is metabolically stable. This property allows them to be used for silencing with antisense oligonucleotides [90]. We designed 11 nucleotides long antisense olinscide (5′-ACACCGACGAC-3′—ICER-11) from the *I. purchasi* 28S ribosomal RNA gene, respectively, and applied (sprayed) them to the target plant (1 mg of DNA per m^2^ of plant leaves). In the groups treated with water, and ICER-11, we observed deaths of 8.06, 41.64%; 12.25, 67.08%; 18.44, 83.02%, respectively, on the 4th, 7th, and 10th days after treatment (ICER-11 vs. control: χ^2^ = 29.04, df = 1, *n* = 200, *p* < 0.001; ICER-11 vs. control: χ^2^ = 61.01, df = 1, *n* = 200, *p* < 0.001; ICER-11 vs. control: χ^2^ = 81.93, df = 1, *n* = 200, *p* < 0.001). Relative to controls, larval mortality in the ICER-11 group was accompanied by a significantly decrease (6.4-fold) of the target gene expression on the 7th day [94].

Insecticide resistance is a serious issue that might be solved by using olinscides to slow it down. Short single-stranded snippets of highly conserved portions of the insect host genes have been used to demonstrate that the rate of possible mutations that might alter the target genes is extremely low [57]. Using oligonucleotides based on relatively conservative portions of functionally significant genes, such as ribosomal genes, we may be able to slow down the evolution of pesticide resistance even if we are unable to stop the genetic mechanisms that contribute to it [90,91,92,95].

## 3. Superfamily Aleyrodoidea

Representatives (whiteflies) of the superfamily Aleyrodoidea do not differ much from their closest relatives from suborder Sternorrhyncha in their harmfulness. They are notorious pests known throughout the world. They infect plants of food, industrial, and ornamental value, causing losses of hundreds of millions of dollars [96]. Most serious whitefly outbreaks occur in greenhouses. The high density of planting and the creation of conditions favorable for plants in greenhouse complexes, similar to the climate of the zones from which they evolved, contribute to the active development and reproduction of these pests. Like related taxa, Aleyrodoidea are involved in the spread of pathogens [96,97].

The cultivation of some plants due to the spread of the whitefly is sometimes laborious, involves the application of a large number of chemical insecticides, and is not always combined with biological control methods. Although many species are successfully controlled with the help of parasitoids [98], this method is not always effective or justified. It has been estimated that the application of *Encarsia formosa* Gahan to control *B. argentifolii* on *Euphorbia pulcherrima* Wild, ex. Koltz is not commercially justified (an increase in labor costs of 56% and material investments of more than 30%) compared with the use of imidacloprid and will become possible only when wasp production becomes more reliable and cheaper [97].

Often, a heavy whitefly infestation causes the fruits of the plants to not ripen, which makes them completely unsuitable for sale or for further seed production. Usually, when a pest appears on food crops, it makes the plants weaker, the yield decreases, and the taste of the fruit suffers. With a systematic attack by whiteflies, plants die, losing energy [99]. Sooty fungi, as secondarily attached pathogens, contribute to the growth of economic losses from the vital activity of other Sternorrhyncha species, but it is in combination with whiteflies that they have the most powerful effect, completely affecting fruits covered with honeydew and contributing to their spoilage.

Since the amount of honeydew released by insects directly depends on plant chemistry, the critical economic threshold of harmfulness for each species will be different. In particular, based on the data on greenhouse whitefly (*Trialeurodes vaporariorum* Westw.), for cucumber, this number is around 60 individuals per leaf, and for tomato it is around 10 [100].

Most whitefly species are polyphagous; for example, *B. tabaci* has more than 500 hosts [96]. However, this is not the only reason why whitefly control is of particular importance. Excessive economic casualties worldwide among fruit and vegetable crops and ornamental plantings are due to development *B. tabaci* vector viruses [101,102]. *B. tabaci* transmits five genera of plant viruses to host plants [30]. In particular, the differentiation of *B. tabaci* biotype biotype “B” insect pests capable of infecting a larger number of host plants (approximately 600 species) has resulted in the infection of previously healthy agricultural plant species with geminiviruses. Agricultural crops of the European space turned out to be able to be affected by viruses carried by the whitefly [101,102].

Representatives of the superfamily Aleyrodoidea have developed and carry various resistance mechanisms the insecticides used in their control [103]. To protect plants from insect pests from the superfamily Aleyrodoidea, the following classes of insecticides are used: organophosphates (OPs), carbamates, and pyrethroids, all of which have a wide spectrum of action and unfavorable ecotoxicological characteristics [103,104,105,106,107]. Their widespread use has led to strong selection pressure due to their long-term, frequent, and irrational use, and whitefly populations have developed resistance to them worldwide [108]. According to numerous studies, *B. tabaci*, a member of the superfamily Aleyrodoidea, the most economically significant insect pest has been shown to affect a wide host range, is rapidly growing in the world population, and is capable of developing a strong resistance to insecticides [109]. Despite the presence of several strains in the population, all of them demonstrate the development of resistance [103]. For example, according to studies by scientists from Turkiye, all the studied organisms showed high levels of resistance to pyrethroid substances (from 57 to 360-fold) and OPs (from 20 to 310-fold) [110]. According to scientists from Crete, *B. tabaci*, originating from a greenhouse, showed the highest resistance to all insecticides: at LC_50_, resistance factors were 730-fold for imidacloprid, 80-fold for α-cypermethrin, 58-fold for endosulfan, 23-fold greater for bifenthrin, and 18-fold for pirimiphos-methyl [111]. Excessive use of insecticides has declined to a greater level of susceptibility in various populations of the Trialeurodes genus, in particular *T*. *vaporariorum* [112]. In total, there are 62 species of this genus in the world [113].

More details about the features of the structure and life cycle of whiteflies can be found using the example of bay whitefly *Trialeurodes lauri* Signoret, a species that is widespread in the Mediterranean. Members of the genus Trialeurodes are of tropical and subtropical origins. It is known that this species was described by Signoret (1882) on laurels in Greece, while it was noted that the appearance of the species causes severe infection in Crimea (Nikita Botanical Garden, personal communication) and Turkiye [98,114]. Relatively recent records come from the British isolate specialists, who found seriously damaged strawberries while importing wreaths for Christmas from Turkiye [98]. *T. lauri* has a wide distribution in the Mediterranean region, where it has been reported in Croatia [115] and in the former Yugoslavia. It may also have developed in Montenegro, but no published evidence has been found [116].

The main host plant is *Laurus nobilis* L. On it, the pest infects both surfaces of the leaf blade. Development can also occur on *Arbutus andrachne* L., *Arbutus unedo* L. [117], and *Myrtus communis* L. [116,118]. Almost all plants parasitized by *T. lauri* (i.e., *A. andrachne*, *A. unedo*, *L. nobilis*) are evergreens that inhabit the landscapes of the southern territories of Europe and the Mediterranean forests with an altitudinal zone of 500 m above sea level. Such plants renew their leaf vegetation in March–May in the spring [98].

The life cycle of whitefly representatives is characterized by high reproductive capacity and a destructive lifestyle, which determines their ability to cause serious economic and agricultural damage. Morphological characteristics unique to whiteflies are the winged adult stage, all of whose integuments, including the wings, are covered with a wax coating, as well as the sessile stage (so-called “pupa”), the appearance of which is radically different from other stages of insect development [119]. Adult bay whiteflies appear en masse on plants in April–May and lay their eggs on the young foliage of host plants. The ovipositions on the bottom surface of the leaf are arranged in groups so that their longitudinal axis is parallel to its surface [98]. Eggs hatched develop in a short time into early fourth instar nymphs, which enter diapause until the beginning of the next spring, after which they pass into the adult stage [120]. More than half of the diameter of the nymph is usually a wax cover with horizontally protruding wax filaments. Periodically, *T. lauri* is capable of spawning hundreds of nymphs on the area of the blade of one leaf, which is also an invasion.

The main way of distribution of the whitefly, in particular *T. lauri*, is through the transportation of planting material in international trade [116]. An important factor contributing to these processes is global climate change [116,121]. Climate change is a complex, multifactorial process that includes changes in temperature, increased temperature, increased droughts, and more frequent storms. It is anticipated that climate change will lead to the selection of insects with a short breeding period, high fecundity, and high survival rates [122]. Whiteflies, with their life cycle features, pose a threat to the agricultural, ornamental, and plant areas (sectors) of the economy. The complex interplay between abiotic and biotic factors makes it difficult to predict overall consequences [123]. Climate change is expected to cause the intensive use of insecticides [124].

However, recent findings show that human-related activities, such as the use of pesticides and other chemicals, have contributed tremendously to the effects of climate change via the emission of greenhouse gases from these chemicals [54,125,126]. As a solution, we propose the use of oligonucleotide insecticides [57] with a zero (or minimal) carbon footprint for insect pest control. The modern solid-phase synthesis of oligonucleotide insecticides (olinscides) on DNA synthesizers using phosphoramidites does not lead to the accumulation of greenhouse gases: carbon dioxide, nitrogen oxide, methane, and ozone. We designed the eleven nucleotides long antisense olinscide (5′-ATGATCCTTCC-3′—Trial-11) from the *T. lauri* 28S ribosomal RNA genes, respectively, and applied them to the target plant (1 mg of DNA per m^2^ of plant leaves). Our pioneer experiments performed on the Southern Coast of Crimea show that olinscide Trial-11 (5′-ATGATCCTTCC-3′) causes up to 82% mortality in *T. lauri* larvae on laurel on the 14th day (unpublished data).

## 4. Superfamily Psylloidea

Psyllids, often known as jumping plant lice, are members of the suborder Sternorrhyncha and make up the superfamily Psylloidea. There are over 4000 known species of psyllids, with at least as many more unrecognized [127]. While not yet complete, David Ouvrard’s Psyl’list online database intends to compile all taxonomic data along with related data, such as host plants and geographical details [128]. Host plants are mentioned in Psyl’list in the same manner as they are stated in the source articles, since the material in Psyl’list was obtained from scientific publications. As of August 2022, the database had records for 3702 different psyllid species. The database provides a general overview of host-plant association patterns in the Psylloidea. This database lists the following seven families as belonging to the superfamily Psylloidea Latreille: Aphalaridae, Carsidaridae, Calophyidae, Homotomidae, Liviidae, Mastigimatidae, Psyllidae, and Triozidae. Twenty family-group names and 28 genus-group names are synonymized, and the families Aphalaridae, Liviidae, and Psyllidae are redefined [129].

Psyllids are closely related to aphids, scale insects, and whiteflies, and only consume plant sap. However, unlike these other insects, they are often very specialized in terms of the plant species on which they develop. [128,130]. The majority of host plants are classified as eudicots (such as the *Fabaceae* Juss., *Myrtaceae* Juss., and *Sapindales* Juss. ex Bercht. and J.Presl.), with the *Magnoliales* Bromhead coming in second. Only a few species are connected to conifers and monocots [131]. Except for Antarctica, all biogeographical zones of the planet are home to psyllids. In the tropics and mild regions, their differences are most noticeable. However, the least studied faunas, the Afrotropical and Neotropical biogeographical domains, are probably particularly species-rich [132]. Jumping plant lice on plants produce abundant nectar, cover frequent wax secretions (burning the canopy of damaged plants), and inject toxic saliva (causing necrosis, teratogen or bile), and are ultimately responsible for the transmission of many pathogens, mainly bacteria, especially phytoplasmas, to plants [133].

Potato (*Solanum tuberosum* L.), tomato (*Solanum lycopersicum* L.), tamarillo (*Solanum betaceum* Cav.), eggplant (*Solanum melongena* L.), and pepper (*Capsicum* spp.) are the primary agricultural plants on which psyllids parasitize [134]. They also parasitize on carrot (*Daucus carota* L) [135] *Citrus* spp. and *Murraya* spp. [136,137,138], pear (*Pyrus* spp.) [139], apple (*Malus domestica* Borkh.) [140], and other important crops. Damage by psillides affects a variety of ornamental and landscape plants, including species of *Eucalyptus*, *Acacia*, *Vachellia*, *Buxus*, *Schinus*, *Ficus*, and *Lauru*s. Some psyllids are employed or under consideration for the biological control of invasive weeds, including the Brazilian peppertree (*Schinus terebinthifolia* Raddi) or melaleuca (*Melaleuca quinquenervia* (Cav.) Blake) in Florida, the velvet tree (*Miconia calvescens* DC.) in Hawaii, and Japanese knotweed (*Fallopia japonica* Houtt.) in Europe. [141,142,143]. Usually, the number of insects from the Psylloidea superfamily is controlled using chemical insecticides such as abamectin, endosulfan, and imidacloprid [144], and thiamethoxan, bifenthrin, carbosulfan, chlorpyrifos, deltamethrin [145,146], spiromesifen, and biological insecticides such as spinosad [147,148].

Psyllid *Macrohomotoma gladiata* Kuwayama is another species of phytosanitary concern due to its ability to coat leaves with wax and honeydew exudates that can cause direct and secondary damage to host plant branches [149]. In turn, *D. citri* resistance to various classes of insecticides has developed [150]. For instance, *D. citri*, along with other pest-borne citrus diseases, has become a problem in and of itself in Pakistan as a result of the indiscriminate and unsustainable use of pesticides in plantations [151]. One of the primary causes driving the search for new pesticides with novel or underutilized modes of action is the continual development of pest resistance to insecticides [152]. At least 489 distinct insect species have developed resistance to 400 different chemicals, making insecticide resistance a severe global issue [153]. Therefore, the development of new classes of insecticides is constantly required. DNA-based antisense short single-stranded oligonucleotides (olinscides) were effective against the Sternorrhyncha suborder species [91,92,154,155,156]. We designed two antisense oligonucleotides designed to inhibit ribosomal 5.8S RNA gene expression. On laurel plants infected with *T. alacris* larvae (1 mg DNA per m^2^) of plant leaves, these oligonucleotide fragments Alacris-11 (5’-CCACCGGGTAG-3’) and Laura-11 (5’-GACACGCGCGC-3’) were applied. On the 9th day after treatment, we observed larval death. The mortality in the water-treated control group was 8.68 ± 4.9%, while the mortality in the random oligonucleotide (5’-CTGACTGACTG-3’)-treated group was 14.37 ± 3.25%. The mortality of bay sucker larvae following treatment with Laura-11 antisense fragment was 72.39 ± 6.48% and Alacris-11 antisense fragment 71.02 ± 5.21% [93].

It is noteworthy that the chemical synthesis of oligonucleotides has now been widely utilized in the fields of molecular biology and medicine for the production of primers, probes, diagnostics for various diseases, gene assembly, vector introduction, genome sequencing, gene editing, gene modification, and drug development based on antisense technologies [157,158]. The solid-phase method based on phosphoramidite synthesis is the most popular and commonly applied technique for the synthesis of oligonucleotides in practice [159]. It allows one to get the required sequence of oligonucleotides quickly, efficiently, and with high productivity and purity. However, this effective method has a sufficient number of different problems. It requires a large number of reagents to form a small number of oligonucleotide chains. Additionally, the method is based on the use of a large amount of porous glass (CPG), which is a rather expensive component. The high cost of automated nucleic acid synthesizers also makes the production process for the synthesis of oligonucleotide sequences expensive. An alternative to the method is liquid-phase synthesis [160]. It is important to note that by moving the synthesis of oligonucleotides to chemical reactors, automatic synthesizers become superfluous, the production process does not require an expensive solid phase carrier (CPG), and the costs of the production process can be significantly decreased while the productivity of synthesis is increased. Calculations show that oligonucleotide costs can be reduced by a factor of 100 using liquid-phase synthesis.

## 5. Superfamily Aphidoidea

The superfamily Aphidoidea includes about 5000 species, the main part of which belong to the tribe Aphidinae (2579 species) [161]. Aphids are small insects, and their does not usually exceed a few millimeters. In their life cycle, wingless (intended for active parthenogenetic reproduction) and winged (responsible for settlement and sexual reproduction) forms alternate. Sexual dimorphism in this taxon is not clearly expressed; the wings mainky differ [162]. A huge number of economically significant species from this taxon are known for their often-fatal impacts on key agricultural crops, including corn [163], wheat [164], cotton [165], barley and sorghum [166], rice [167], and others.

Representatives of Aphidoidea, in addition to widespread distribution around the globe, have another serious trump card in the arsenal of their evolutionary adaptations: unprecedented reproductive ability [168], a high reproduction rate, a short development time from the larva stage to the semi-mature individual (on average, about 5 days), and the presence of a series of parthenogenetic generations, and live birth, which, for the aforementioned Coccoidea, is the exception rather than the rule. All of them are characterized by feeding with phloem juice [169], which became possible thanks to specific saliva proteins that prevent the normal reaction of the plant to damage. For example, ref. [170] demonstrated that *Megoura viciae* Buckton saliva induces dispersed forisomes in order to suppress clogging of the sieve tubes in *Vicia faba* L.

The combination of physiological and biochemical adaptations of aphids to nutrition makes them superpests that cause a serious blow to the economies of agricultural regions of the world, reaching from tens of millions to a billion dollars annually [171]. Losses associated with the appearance in 2013 and the spread of the sugar cane aphid *M. sacchari* in North America and Mexico reached almost 50% of the sorghum crop at a level of 98% of crops [10]. The alternation of angolocyclic and holocyclic stages of population development allows aphids to simultaneously produce powerful outbreaks of numbers and actively increase resistance to chemical insecticides. Thus, for example, in the USA, the resistance of the *A. gossypii* to organophosphates and pyrethroid was recorded as early as 1992 [172]. Despite the growing resistance, large farmers are forced to continue using chemical means of protection, combining them with biological methods [173] and botanical insecticides [174,175,176].

In addition to the fact that aphids cause enormous damage to world agriculture, floriculture and ornamental gardening also suffer from them [177,178,179]. Roses, chrysanthemums, tulips, oleanders, jasmine, and conifers are crops used for landscaping in recreational areas. It is obvious that the use of chemical insecticides to control aphids is associated with risks to human health and the well-being of other components of the environment [180].

A more conscious approach to population regulation was founded on increasing the density of natural enemies of aphids, for example, *Coccinella septempunctata* L. [181]. An important aspect of the effectiveness of the *C. septempunctata* against aphids is the spatial configuration of the landscape. It has been proven that landscapes with less than 4% non-crop habitat are not able to maintain the necessary level of protection against aphids [182]. The predatory potential of larvae and imago varies; adults eat at least two times more aphids per day than larvae [183]. In general, during the stage of development, imago kills more than 1400 aphids [183].

There are monitoring systems that involve monitoring pests on crops, but they are often inaccurate. After all, it is extremely difficult to predict outbreaks of aphid abundance due to the multifactorial nature of the event: current abundance, fertility, mortality, migration coefficient, temperature, and humidity [184,185].

The largest tribe of aphids from the Aphidinae subfamily is the Macrosiphini tribe, which includes many pests of agricultural and ornamental plants, among which are pests of economically significant flower crops: roses (29 species), chrysanthemums (33 species), tulips, and lilies (more than 11 species) [186,187]. There are a significant number of mono- and oligophages in this tribe of pests of flower crops. A typical species characterized by oligophagy is the chrysanthemum aphid *Macrosiphoniella sanborni* Gillette. Despite the presence of natural enemies *Aphidius absinthii* Marshall, *Toxares shigai* Takada [188], *Toxares macrosiphophagum* Shuja-Uddin [189], and *Ephedrus niger* Gautier, Bonnamour, and Gaumont [190], biological control with their help is ineffective, because the ranges of these species are limited. In this regard, the development of alternatives to chemical insecticides and biological methods of controlling the number of chrysanthemum aphids is relevant.

Chrysanthemum aphid *M. sanborni* is the first pest of flower crops studied by our scientific group from the point of view of olinscide testing. Chrysanthemums are among the four most popular flower crops in the world [191], so commercial losses in the cultivation of this plant often become significant and affect the welfare of exporting countries [192]. *M. sanborni* is a holocyclic species native to Asia [193]. It primarily affects young shoots and chrysanthemum buds, leading to a reduce in the quantity of flowering shoots.

A large number of publications are devoted to the topic of the adverse effect of chemical insecticides on useful insects [194,195,196]. This is a large problem that agrarians are facing and this needs an effective solution. After all, with the disappearance of beneficial insects, especially pollinators, the management of agrocenoses will become even more difficult, and their existence will be in question [197]. Studies indicate the toxicity of most chemical pesticides, both for *Apis mellifera* L. [198] and for solitary bees [199]. Moreover, this action is systemic and tissue-specific, causing irreversible changes in the organism of bees and their rapid disappearance. For example, a change in the thermal and olfactory behavior of bees is recorded under the influence of pyrethroids and strobilurins [200]. The modulation of head, midgut, and abdominal various enzymes under the influence of imidacloprid, which are metabolism and immunity, is involved in the detoxification of pesticides and compensates for high levels of oxidizing substances [198]. Such significant changes are also confirmed by the transcriptomic data [201]. One of the studies reports that in 2002, in Uruguay, against the background of the extinction of bee families, monitoring studies began, during which the presence of imidacloprid and fipronil was detected in deserted hives [202].

For example, there is a worthy alternative to chemical pesticides, characterized by selectivity of action, relative affordability, and safety for non-target organisms: olinscides (DNA insecticides)—unmodified antisense DNA oligonucleotides [91,203,204]. Earlier studies [205] indicate the absence or presence of a short-term effect lasting less than a week on *Quercus robur* L., *M. domestica*, and *Triticum aestivum* L. [203]. The principle of their action is based on blocking the expression of functionally important genes (e.g., anti-apoptosis, ribosomal, etc.) in insect pests on the basis of antisense interactions. Notably, higher insect pest selectivity was demonstrated, consisting of the absence of an effect when replacing one nitrogenous base out of eleven. This does not allow for the olinscide to work effectively, and substantially decreases the insecticidal effect (unpublished data).

We have developed olinscide Macsan-11, which has an affinity for the 5.8S rRNA gene and has shown promising results. The treatment of plants affected by the pest resulted in 67.15 ± 3.32% mortality (χ^2^ = 448.8, *p* < 0.001, *n* = 1100, dF = 1) on the 7th day after a single treatment with a water solution at a concentration of 100 ng/L and mortality 97.38 ± 2.49% (χ^2^ = 360.8, *p* < 0.001, *n* = 1100, dF = 1) on the 7th day after twice-daily treatment with the same solution [206]. The introduction of the group with double treatment was due to the following observation: viviparous females, unlike larval forms, had greater resistance to the action of Macsan-11. It is most likely that the insecticidal load on newborn aphids was extremely low and that the second treatment made it possible to cover a larger number of vulnerable individuals.

## 6. Conclusions

Without exaggeration, we can say that the suborder Sternorrhyncha belongs to one of the most serious groups of insect pests. These types of insect pests are invisible superplunderers of plant vitality that cause significant damage to the global economy and significantly reduce plant productivity. Experts agree that at present there is no effective replacement for insecticides. Olinscides currently possess unique characteristics that make them a promising direction that can compete with chemical insecticides. Olinscides have great potential to be used as safe and effective insecticides against hemipterans. We investigated olinscides as a part of simferogenomics (Greek: συμφερο—usefulness), which uses antisense oligonucleotides as a tool for the selective regulation of insect pests at organismal and supraorganismal levels, benefiting agriculture and forestry. The use of oligonucleotides as insecticides is very attractive, as they act at the molecular level according to perfect complementarity principle, which allows them to be quickly biodegradable in nature and selective in action and compared to most chemical insecticides and RNAi approach. There are serious reasons as to why olinscides are more advantageous in comparison with RNA preparations, like SIGS and HIGS, and are better utilized on a large scale against insect pests from suborder Sternorryncha. First, in both cases, using DNA/RNA synthesizers or a genetic engineering approach, short (~11 nt long) insect-specific olinscides are more affordable to be produced compared to relatively long and ‘expensive’ double-stranded RNA fragments. Second, very short 11 nt long pest-specific antisense oligonucleotides have their precise target gene in target organisms, while relatively long dsRNAs are diced into very short non-target siRNAs, and thus non-target genes may be silenced in non-target organisms [207]. Third, the hydrolysis of DNA is much less facile than the hydrolysis of RNA because of the presence of the 2’-H in pentose ring [208]. Thus, oligonucleotide insecticides will be more stable than RNA preparations in the environment and cause a prolonged insecticidal effect before being biodegraded. The affordable and widespread commercial synthesis of nucleic acids also makes olinscides a promising class of insecticides for plant protection. In our opinion, olinscides are the key that will unlock the door of the control of insect pests from suborder Sternorrhyncha and occupy its niche in the insecticidal market, gradually expanding its influence on other groups of insect pests. To date, the development of molecular genetics with genomic databases has been the best way to promote the spread of olinscides. In plant protection, we are approaching the point where the selectivity of an insecticide will be as highly valued as its effectiveness, and olinscides [209] make this possible.

## Data Availability

Not applicable.
